# Local Culture and Community Through a Digital Lens: Viewpoint on Designing and Implementing a Virtual Second Look Event for Residency Applicants

**DOI:** 10.2196/44240

**Published:** 2023-09-11

**Authors:** Jaclyn M Martindale, Rachel A Carrasquillo, Scott Ireland Otallah, Amber K Brooks, Nancy Denizard-Thompson, Emily Pharr, Nakiea Choate, Mitchell Sokolosky, Lauren Doyle Strauss

**Affiliations:** 1 Wake Forest University School of Medicine Winston-Salem, NC United States; 2 Department of Neurology Atrium Health Wake Forest Baptist Winston-Salem, NC United States; 3 Department of Anesthesia Atrium Health Wake Forest Baptist Winston-Salem, NC United States; 4 Department of Internal Medicine Atrium Health Wake Forest Baptist Winston-Salem, NC United States; 5 Department of Emergency Medicine Atrium Health Wake Forest Baptist Winston-Salem, NC United States

**Keywords:** medical education, graduate medical education, residency application, virtual interviews, match, recruitment

## Abstract

**Background:**

The COVID-19 pandemic altered how residency interviews occur. Despite 2 years of web-based interviews, these are still perceived as inferior to in-person experiences. Showcasing a program and location is critical for recruitment; however, it is difficult to highlight the program’s location and community digitally. This article presents the authors’ viewpoints on designing and implementing a virtual second look for residency applicants.

**Objective:**

Our objective was to host a web-based event to feature the benefits of living in Winston-Salem, North Carolina, for residency applicants, enhance recruitment efforts, and ensure a successful residency match. The goal was to cover topics that interested all applicants, highlight how Winston-Salem is a special place to live, involve current residents, and engage community members.

**Methods:**

Three programs–child neurology, neurology, and family medicine were chosen for a pilot virtual second look. All residency program directors’ were asked to recommend community contacts and help identify residents and faculty who may serve as content experts on one of the topics in the panel discussions. A total of 24 community leaders from restaurants, venues, schools, and businesses were contacted, and 18 agreed to participate. The panel discussions included living in and raising a family in Winston-Salem, experiencing Winston-Salem arts and music, where to eat and drink like a local, and enjoying sports and outdoors in the area. The 2-hour event was hosted on Zoom. Postevent feedback assessments were automatically sent to each registrant through Research Electronic Data Capture (REDCap). This study was deemed exempt from Wake Forest University Health Sciences institutional review board review (IRB00088703).

**Results:**

There were 51 registrants for the event, and 28 of 48 registrants provided postevent feedback, which was positive. The authors found in the MATCH residency results that 2 of 2 child neurology positions, 4 of 6 adult neurology positions, and 1 of 10 family medicine positions attended our second look event. One adult neurology resident who did not participate was an internal candidate. All respondents agreed or strongly agreed that the session was valuable, well organized, and met their expectations or goals. Furthermore, all respondents gained new information during this web-based event not obtained during their interview day.

**Conclusions:**

The virtual second look event for residency attendees featured the benefits of living in Winston-Salem, and the perspectives of current residents. Feedback from the session was overall positive; however, a top desire would be devoting more time for the applicants to ask questions directly to the community leaders and our resident trainees. This program could be reproducible by other institutions. It could be broadened to a graduate medical education–wide virtual second look event where all medical and surgical programs could opt to participate, facilitating an equitable opportunity for prospective applicants.

## Introduction

### Background

In March 2020, the COVID-19 pandemic disrupted typical medical education operations. Only 6 days after declaring a global pandemic, the Association of American Medical Colleges (AAMC) recommended ceasing all medical student clinical rotations [1,2]. In addition, by May 2020, AAMC announced the recommendation of web-based interviews for the 2021 match season [3].

Social media swiftly became an essential focus in digital recruitment, with a significant rise in residency program social media accounts during the pandemic [4-6]. Programs used social media to offer glimpses into the day-to-day life of residents, provide resident spotlights, share resident wellness initiatives, and advertise web-based open houses. In addition, programs developed web-based events before and during interview dates. These often featured program leaders and residents, video overviews of the community, or hospital tours to highlight the lifestyle and location in their regions.

Social interactions, work-life balance, and location culture are priorities for applicants [7]. Applicants felt social media created transparency, relayed values, and potential fit of the program. However, a survey of child neurology residency programs suggested web-based interviews were still perceived as inferior to in-person experiences. Additional challenges occur for programs in smaller or midsize towns that applicants may be less familiar with or have never visited. Showcasing the program and location becomes more critical for recruitment; however, it is difficult to highlight the program’s location and community in the web-based setting [8,9].

Wake Forest University School of Medicine (WFUSOM), headquartered in Winston-Salem, North Carolina. Since the founding of the medical school in 1902 and the medical center in 1923, Wake Forest has grown into a nationally recognized academic medical center and health care system. The catchment area is a 24-county region, including western North Carolina and Southwest Virginia, extending to Tennessee and West Virginia. The hospital is the largest tertiary care center for the Piedmont region of the Southeast. However, Winston-Salem is a midsize city with an estimated population size of 250,000 [10].

The AAMC recently recommended a third season of web-based recruitment [11]. However, how do programs adequately reflect the culture and locale through a digital lens? To address this gap, we created a virtual second look event for our institution’s child neurology, neurology, and family medicine applicants. This article presents the authors’ viewpoints on designing and implementing a virtual second look for residency applicants.

### Objective

The Graduate Medical Education (GME) Committee at WFUSOM recognized that there was a need to feature the benefits of living in Winston-Salem to applicants. Therefore, the GME invited one program’s leadership (authors LDS and JMM) to plan a web-based pilot event with 3 residency programs. Our aim was to host a successful web-based event to feature the benefits of living in Winston-Salem for residency applicants, enhance recruitment efforts, and ensure a successful residency match. Each program has hundreds of applicants, so the event would need to be scalable to a larger potential audience in the future if the initial session was a success. The web-based offering would cover topics that interest applicants, highlight how Winston-Salem is a special place to live, involve current residents from various programs, and engage community members.

## Methods

### Program Development

LS, an executive member of the GME and program director of the child neurology residency, presented to the GME in the August of 2021 to propose objectives, dates, and timelines. All residency programs were invited, and the first 3 programs that responded were included in the pilot- child neurology, neurology, and family medicine. A planning committee was formed, including authors JMM, NC, and LDS and GME specialist Mikell White. Four themes were identified from collective feedback following a presentation at a GME meeting with program directors and from discussions with adult neurology and child neurology residents (1) living in and raising a family in Winston-Salem, (2) experiencing Winston-Salem arts and music, (3) where to eat and drink like a local, and (4) enjoying sports and outdoors.

A planning committee was formed with GME and program leaders. As a result, a date was chosen on a Friday in February 2022 to not conflict with interview dates and to reflect a typical working day for community members. Additionally, this date was chosen to allow programs to have submitted their rank list yet allow applicants to have time to adjust their decisions. Logistically, this also allowed enough time to finalize an agenda and attendees from the community.

Program directors from all residency programs were asked to recommend contacts for the community and help identify residents and faculty who may serve as content experts on one of the topics in the panel discussions. The event was free. None of the participants or community partners were compensated for their time.

Authors LDS and JMM partnered with a local tourism company to identify a speaker to introduce the Winston-Salem tourism industry and develop contacts for the community leaders and organizations. A final agenda, including panelists, was reviewed for content with our GME executive team. The GME had already held a successful web-based Diversity and Inclusion event led (led by authors AKB and NDT) for invited underrepresented minorities, so careful consideration was made not to overlap in content (Multimedia Appendix 1). Our Accreditation Council for Graduate Medical Education (ACGME)-designated institutional official and associate dean of GME for the WFUSOM (author MS) were invited to present about the GME programs and shared resident resources.

A total of 24 community leaders from restaurants, venues, schools, and businesses were contacted, and 18 agreed to participate as panelists. Individual meetings were arranged with each community leader to share the prospective resident interests and help narrow the focus of their discussion during the panel session. This connection helped answer questions about the target audience and why we recruit them. The authors also used this time to propose questions and learn about opportunities in the community for residents. Consent was received from all community leaders to include their organization’s brand in our advertisements and communications with invited applicants. This process took several weeks and occurred between November 2021 through January 2022. The authors added faculty moderators (n=4) from the school of medicine and resident panelists (n=4) from neurosurgery, neurology, child neurology, and family medicine residency programs.

A web-based brochure included photos, bios, social media contacts, and website resources. This brochure was an aid for the moderators to learn more about panelists and a guide shared with all applicant attendees to learn more about the community. The brochure was reviewed with all participants before being shared with applicants.

The session (Textbox 1) was designed to be short and fast-paced over 2 hours to optimize applicant attendance. The session was hosted within the Zoom videoconferencing platform with 2 GME program coordinators available for technical support. The panelists were expected to check in 15 minutes before their panel discussion and stay for the duration of their discussion, keeping their commitment time to less than 1 hour. A full agenda was shared with moderators, including a full script of anticipated questions for panelists. Panelists received their questions over email the week of the event to allow them to anticipate their area of focus. Each panelist received guidance on the length of time per question and was given 1-2 questions.

**Outline of the virtual second look program.**

**Welcome presentation (30 minutes)**

- Introduction: Child Neurology Program Director
- Welcome: Wake Forest University School of Medicine’s Graduate Medical Education Designated Institutional Official
- History of Winston-Salem: Local Visitor Center Director of Marketing and Communications
- Schedule introduction: Child Neurology Associate Program Director

**Panel 1: Living in and raising a family in Winston-Salem (30 minutes)**

- Panelists: family medicine resident, child neurology resident, resident spousal association leader, local gymnasium president, local sports chief executive officer, city’s recreational special events coordinator
- Moderator: Adult Neurology Program Director

**Panel 2: Experiencing Winston-Salem arts and music (20 minutes)**

- Panelists: conductor of the local medical orchestra and current medical student, local boarding school teacher, local music venue owner and cofounder, University of North Carolina School of the Art director of media relations and communications, local arts council director
- Moderator: Adult stroke neurologist

**Panel 3: Where to eat and drink like a local (20 minutes)**

- Panelists: neurosurgery resident, local restauranteur, local restaurant and bar assistant general manager, local vineyard co-owner, local brewery manager
- Moderator: Child Neurology Program Director

**Panel 4: Enjoying sports and outdoors (20 minutes)**

- Panelists: Family medicine resident, University sports executive associate athletic director, Winston-Salem Open Tournament director, area minor league baseball president and general manager, city parks and recreation special projects coordinator, local district director for community relations
- Moderator: Child Neurology Associate Program Director

Wrap-up: All moderators (5 minutes)

To minimize bias of attendance on the rank list, the adult and child neurology program submitted their finalized rank list before the web-based event. The family medicine program director was not present at the event nor given a list of attendees. Applicants present were not required to turn on their cameras or show their names, although many applicants opted to do so.

A welcome room allowed necessary audio or visual testing to minimize disruption. All moderators and panelists had their cameras turned on with listed names and organizations.

### Advertising Methods

**Figure 1:**
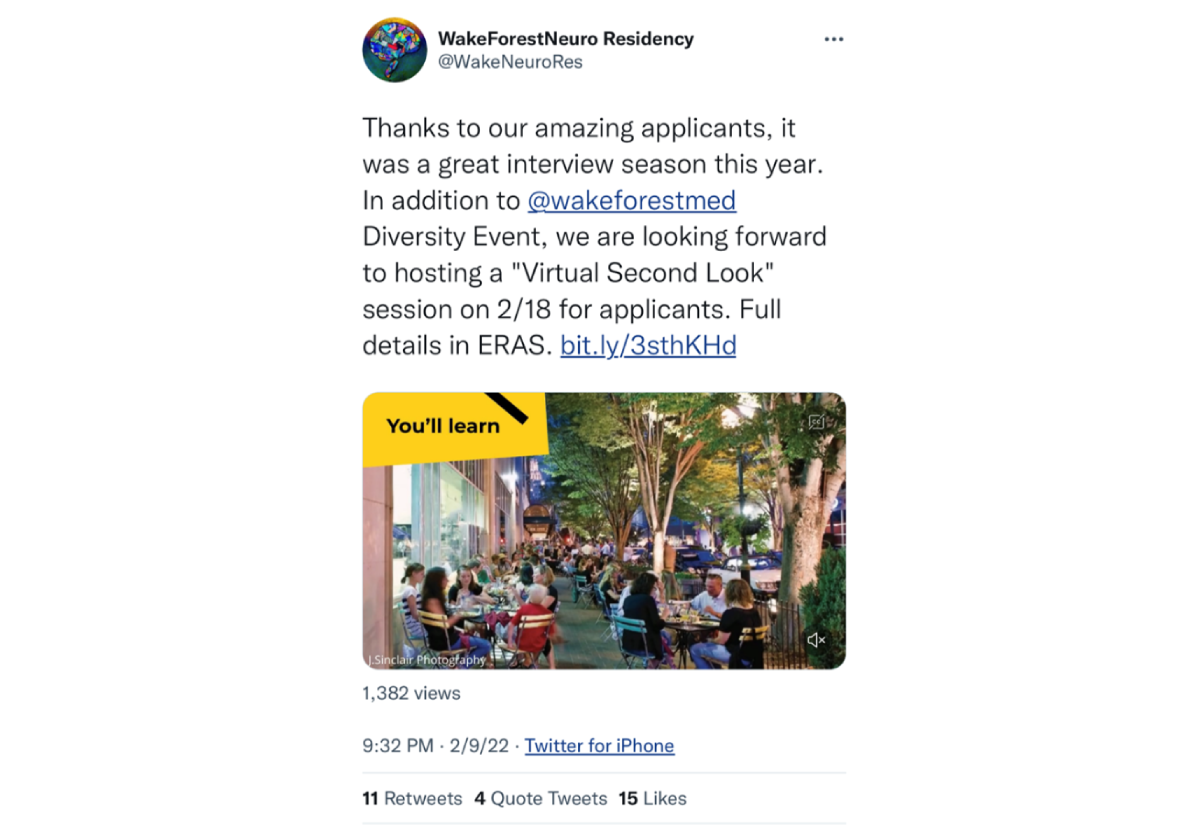
Twitter advertisement of virtual second look event.

Finalizing the panelists and general agenda was necessary before advertising to applicants, which led to a shorter advertisement period. The event was advertised on social media and through an Electronic Residency Application Service (ERAS) communication. On February 09, 2022, an ERAS communication was sent by the program directors of the included programs inviting all interviewed applicants to register. ERAS invites were sent to 50 child neurology, 100 adult neurology, and 167 family medicine applicants. On the same day, a promotional PowToon video created by JMM was posted on the Wake Forest Neurology Residency Twitter account @WakeNeuroRes (Figure 1). This is a combined adult and child neurology social media account run by LS. The post had 11,100 impressions, 15 likes, 4 quote tweets, 11 retweets, 1398 video views, and 8 link clicks. While the family medicine program does have a Twitter account, it was not promoted by social media as this account is not frequently used.

### Assessment Methods

Applicants were emailed a Zoom link for the event upon successful registration through Research Electronic Data Capture (REDCap), which included demographics, which residency program they were applying to, the applicant’s goals for the event, and how the applicant heard about it (Multimedia Appendix 2). Registrants could opt in to have their deidentified data included in the results.

Postevent feedback assessments were automatically sent through REDCap to each of the registrants. The initial feedback request was sent immediately after the web-based event. Automatic weekly reminders to complete the feedback were sent through REDCap for 1 month following the event. Feedback assessments (Multimedia Appendix 3) evaluated the presenters, the content organization, the degree to which expectations were met, and the value of each session and the overall event (Kirkpatrick Level 1). We also evaluated whether new information was gained from the web-based session compared to the web-based interview day (Kirkpatrick Level 2). Applicants were asked to rate each question on a 1-5 Likert scale, with 1=strongly agreed and 5=strongly disagreed. Additionally, applicants provided their top 3 highlights for the events and any suggestions for future improvements.

### Ethical Considerations

This study was deemed exempt from Wake Forest University Health Sciences institutional review board review (IRB00088703).

During event registration, participants were asked “Do you consent to us using your deidentified data for future event planning and research purposes? This will not affect your ability to register or participate in the event.” Registrants could opt-in to have their deidentified data included in the data analysis. Written consent was not obtained. Study results were published in lieu of providing individual subjects with additional information regarding the study. Participants were not compensated.

Confidentiality was protected by collecting only information needed to assess study outcomes, minimizing to the fullest extent possible the collection of any information that could directly identify subjects, and maintaining all study information in a secure manner.

## Results

### Demographics

**Figure 2:**
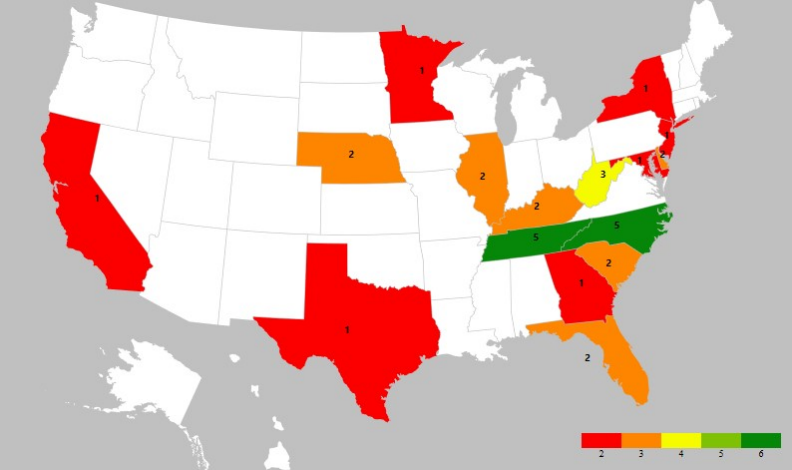
Heat map of registrants.

Between initial advertising and the event date (February 9-18, 2022), there were 51 registrants for the event. Forty-eight consented to have deidentified data included for future event planning and research purposes (Table 1). Most registrants heard about the event through ERAS communication rather than other modalities. Thirty-eight registrants requested a complimentary informational packet about the area through the local visitor center. A heat map using the provided zip codes is included in Figure 2. Most registrations resided in the Southeast; however, some registrants digitally attended nationwide.

**Table 1:** Demographics of registrants (n=48).

Characteristics		Participants, n (%)
**Age (years)**		
	20-30	45 (93.8)
	31-40	3 (6.3)
	41-50	0 (0)
	≥51	0 (0)
**Race**		
	American Indian or Alaska Native	0 (0)
	Asian	12 (25)
	Black or African American	6 (6.3)
	Native Hawaiian or other Pacific Islander	0 (0)
	White	32 (66.7)
	Not disclosed	1 (2.1)
**Ethnicity**		
	Hispanic or Latino	0 (0)
	Not Hispanic or Latino	44 (91.7)
	Not specified	2 (4.2)
	Not disclosed	2 (4.2)
**Specialty of residency application**		
	Child neurology	9 (18.8)
	Family medicine	4 (8.3)
	Internal medicine	1 (2.1)
	Adult neurology	34 (70.8)
**How did you hear about this event?**		
	ERAS^a^ communication	40 (83.3)
	Social Media	1 (2.1)
	Email	14 (29.2)
	Word of mouth	4 (8.4)

^a^ERAS: electronic residency application service.

### Pre-Event Expectations or Goals

Registrants indicated their goals for attending the virtual second look event during registration. Most attendees wanted to learn more about living in the community (45/48, 94%) and learn from current residents about their experience (42/48, 88%). However, learning about the culture of Winston-Salem (34/48, 71%) and restaurants, bars, and wineries (33/48, 69%) were other top priorities for attending the event. Learning about raising a family in the Winston-Salem community was rated the lowest, with only 21% (10/48) of registrants identifying it as a goal for attending the event.

### Postevent Feedback

**Figure 3:**
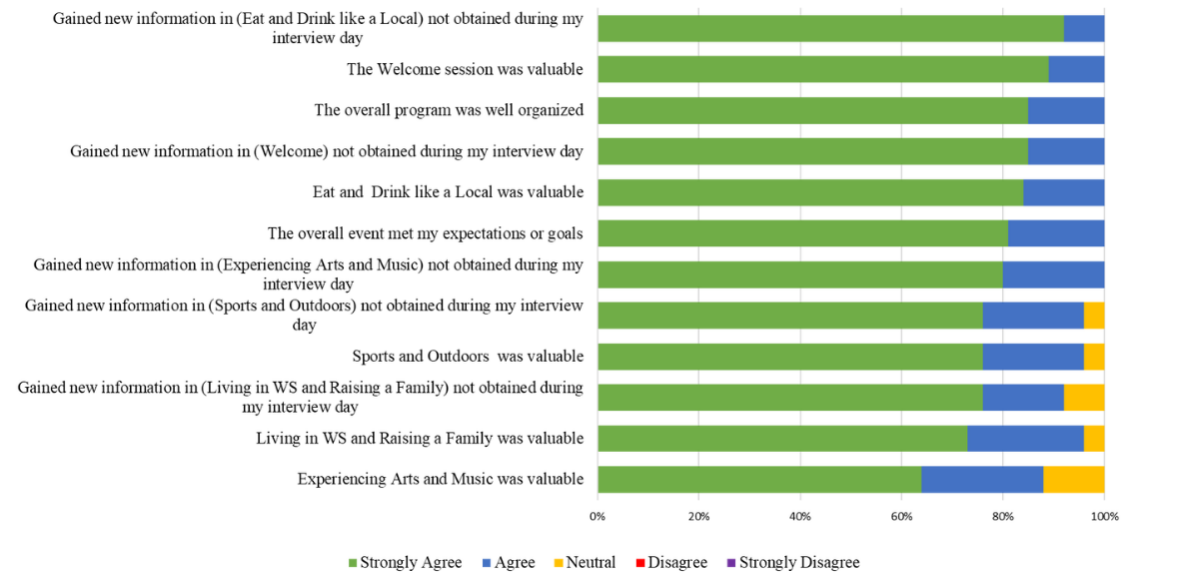
Postevent feedback. WS: Winston-Salem.

We received a postevent feedback response rate of 58% (28/48) of registrants. Overall, the feedback was overwhelmingly positive. All respondents (28/28, 100%) agreed or strongly agreed that the session was valuable, well organized, and met expectations or goals. Additionally, all respondents (28/28, 100%) agreed or strongly agreed that new information was gained during the event about restaurants, bars, arts, music, and the welcome session that was not provided during the interview days. While it still felt valuable, there were more mixed responses to the arts and music panel and the living and raising a family in the area panel (Figure 3). On a scale of 1-10, 1=not effective and 10=very effective, respondents reported a mean of 8.6 level of effectiveness for this event (minimum 7, maximum 10, median 8).

### Qualitative Feedback

Respondents also provided their top 3 highlights and suggestions for future event improvements. JMM developed a codebook of relevant concepts and emerging themes. Two team members coded all comments (JMM and RAC), and coding discrepancies were discussed and resolved. We analyzed the comments using deductive thematic content analysis practices to identify themes and subthemes [12].

The top 3 themes for event highlights included hearing about entertainment or things to do, the living and culture of Winston-Salem, and personal experiences (Table 2). Respondents predominantly highlighted learning about local food and drink options. They also enjoyed personal experiences, particularly from residents. The overall organization, energy, and variety of the event were positive.

**Table 2:** Top themes from respondents on event highlights.

Themes	Supportive quotes
Entertainment or things to do	I appreciated hearing about the local places for food Hearing of the food options, common places to live, and great places to take family should residents have children. I enjoyed hearing from the business owners from the area. I feel like this is unique to Wake Forest and this second look. I appreciated hearing about the local places for food. I am very interested in visiting the llama winery!
Living in and the culture of Winston-Salem	It gave us an idea of where people like to live and also how connected the community is Experiencing the culture of Winston-Salem and hearing about why it is a special place to live Inclusivity of potential schooling, gyms, and other amenities required for residents with families
Hearing personal experiences	The personal experiences of everyone living in Winston-Salem First, it was extremely valuable to hear a resident state the location of a popular area for incoming residents to live in (Ardmore discussion). The different conference sessions, particularly asking current residents questions
Variety of community speakers and topics	The involvement of so many members of the community and their direct point of view. All the people seemed very nice and welcoming. Involvement of people from the community outside of the hospital and school, learning about music and art events and learning about how diverse Winston-Salem is Just hosting this was a standout compared to the other programs. Including community members and talking about life in Winston-Salem in different stages was great and really helped show either how well Wake Forest is regarded or how community-focused the town of Winston-Salem is.
The energy of the event	The interests of everyone who attended allowed for a very organic conversation during presentations. Seeing the people of Winston-Salem being so enthusiastic about promoting the city! I enjoyed everyone’s positive attitude.

The event feedback was overwhelmingly positive. The top theme for feedback was allowing more time for questions. While respondents did have the option to use the chat function for questions, this was not used. Additional improvement themes included increased interaction or breakout sessions, hearing more from residents, and more visuals of the hospital or area (such as web-based tours, photographs, etc).

### Match Results

The authors recognize that the second look event likely attracted a group of applicants more interested in the participating programs. We found in the MATCH residency results that 2 of 2 child neurology positions, 4 of 6 adult neurology positions, and 1 of 10 family medicine positions attended our second look event. One adult neurology resident who did not participate was an internal candidate.

## Discussion

### Principal Results

The recent transition to web-based interviewing has increased interest in developing and expanding web-based content for applicants in various interactive and passive formats. Through this interactive pilot program, we learned that there was high interest from many residency programs to have a GME-led effort on commonly shared needs. Enthusiasm was high for this program even in the first year, making limiting it to only 3 programs in our pilot challenging. Although the content was generalizable to all residency programs, there must be careful consideration of possible challenges in scaling up the number of programs included. A higher volume of attendees could add unanticipated difficulties. In addition, several matches, including the medicine specialty match and fellowship matches, occur throughout the year, so expansion of programming would need to be mindful of timing.

We heard from program directors, residents, and applicants that this was a unique opportunity to add resident and faculty voices that may not be present on an individual program’s web-based interview day. The authors note smaller programs can leverage partnerships with more extensive residency programs. A positive effective resident communicator could help recruit for other residency programs.

Previous in-person and most web-based interview days do not typically include interactive participation from the GME. Our model allowed the GME to share resources directly with applicants. The GME leader spoke about our training institutions’ health, growth, and variety.

Community partners also found the experience to be mutually beneficial. This was seen as a way to help advertise and showcase their opportunities directly to the end user. Most community partners connected us with the owner, the lead of marketing or communication, or the head of programming. Some applicants may choose our health system for residency. However, community leaders saw this as a way to promote our city for future travel or encourage residents to consider our health system again for future fellowship or faculty positions.

Additionally, our faculty reported that it was engaging to see the community partners share their experiences as experts in that content area and cater to a broader audience of varied interests. Program leadership or interviewing faculty may need to learn the answers to some applicant questions because of their interests or community experiences. Of note, although we invited several daycares and preschools, unfortunately, we could not have representation due to the time chosen.

We used REDCap for our invitation and sharing of content, including our web-based brochure and event surveys. REDCap had additional benefits as it allowed registering attendees to sign up without involving our GME staff as it was automated. It also created a list of all those registered that allowed us to follow up on Match and the possible effects of the second look on recruitment. Careful consideration must be given to confidentiality and minimizing the risk of bias on rank list decisions. Options could include finalized rank list submissions for participation in the virtual second look event or blinding the programs from participants.

We had several residents and programs ask to share the content following the event so it could continue in the future as a passive format option to expand our reach. Unfortunately, we did not plan on recording the event and could only make the welcome presentation available. A recording of the event may prove valuable in faculty or resident onboarding.

The majority of the feedback for the sessions was positive. However, some feedback included that the sessions on raising a family and sports and outdoors could have been more valuable. Raising a family was a lower priority for many individuals attending the session initially, likely reflective of different stages of their lives. No specific narrative comments were provided on either of these sessions in the feedback for suggested improvements. The art and music session was also not rated as valuable as the other sessions. In the future, we could modify the types of community members and organizations invited to the sessions to engage and entertain a diverse audience of all ages and stages of life.

The advantages of this event are that it is cost and time efficient for applicants and allows applicants not to take time off from their clinical rotations at their home institution. In addition, having a virtual second look decreases barriers among prospective applicants by giving everyone an equitable opportunity to learn about WFUSOM and the Winston-Salem community.

### Limitations

There were several limitations to this study and event. This is the experience and viewpoints of the authors at an academic center in a moderate city. This may not be generalizable or meet the needs of all institutions. One limitation is the applicant’s ability to access reliable internet and the appropriate technology to attend the event. It also requires time off from clinical duties for the duration of the event. Despite being a high-yield overview of Winston-Salem, it may not highlight the desired aspects for all applicants. Additionally, although virtual second looks facilitate equity, some applicants may prefer to travel to the area to learn more about Winston-Salem.

Regarding the feedback, selection bias may be a limitation. Most of the feedback was positive, and those who enjoyed the event were often more likely to provide feedback. Although narrative comments were requested, there could be more purposeful questioning as to why specific sessions were rated higher or lower. Feedback on the event was requested during a period when programs were submitting their rank lists. While both the adult and child neurology programs had submitted their rank list and the attendee list was not shared with the family medicine program directors, being more transparent with this process may reduce concerns about attendance influencing programs’ rank list. Alternatively, the feedback survey could be sent after the ranking was closed balancing timing to the event to risk the loss of survey participation.

Additionally, those more interested in WFUSOM may have been more likely to attend the virtual second look in the first place. As the child and adult neurology program leadership were involved in creating the event, they could promote the event during their interview season and advertise it on social media. This may have influenced more attendees from these 2 programs over family medicine for the first year. Furthermore, as we waited for a finalized agenda before promoting the event, the advertising period needed longer. This affected the number of applicants and programs we were involved in during our first year of the virtual second look event. Longer and more strategic advertising would increase the reach of recruitment of applicants to attend the virtual second look. Additionally, broadening to a GME-wide virtual second look event where all medical and surgical programs could opt to participate would facilitate an equitable opportunity for prospective applicants.

### Conclusions

The virtual second look event was valuable for child neurology, neurology, and family medicine attendees. It featured the benefits of living in Winston-Salem and the perspectives of current residents. It filled a gap faced in the web-based environment of how to showcase a city and institution. The program has the potential to be expanded to more residency programs at the WFUSOM with the advantages of decreasing barriers among applicants, including the cost of travel and time away from clinical rotations at home institutions.

### Future Directions

Feedback from the session was overall positive; however, a top desire would be devoting more time for the applicants to ask questions directly to the community leaders and our resident trainees. In our first year, we had most of the questions scripted; however, with continued partnership with our community leaders, preparing for open questions in future years would be easier. In addition, time could be set aside for applicants to ask questions directly to the panelists, making the session more interactive for applicants, community leaders, and resident trainees. Future sessions could be recorded so applicants unable to attend could still receive the information and benefits of the session.

Future evaluations of the second look event could assess whether registrants are attending because they are precontemplative, contemplative, and already familiar with the area. Additional areas of interest would include how such an event influences their rank-list decision-making, their decision to attend in-person second look events, and granular feedback about the event itself. Furthermore, comparing the demographics of web-based event attendees to program interviewees, applicants, or the GME demographics as a whole to evaluate the effect of the program on these factors over time would be valuable.

There could be other ways to advertise to increase registration. We learned from our recruitment data that most applicants discovered the event through regular ERAS communication from the programs. Suppose the date is selected far in advance. In that case, it could be shared with applicants during the interview season through web-based interview day discussions, the residency program website, and the GME website. The session could also be scheduled earlier in interview season so applicants could learn more about the Winston-Salem community before ranking their residency choices and help highlight the benefits of our residency program. However, this would need to be carefully balanced bias towards or against applicants while rank lists were open. Last, this program could be expanded to more specialties at the WFUSOM, aiding residency recruitment.

## Appendix

Multimedia Appendix 1 Diversity and Inclusion Event 2022 Agenda.

Multimedia Appendix 2 Pre-event survey.

Multimedia Appendix 3 Postevent survey.

## Abbreviations

AAMC: Association of American Medical Colleges
ERAS: electronic residency application service
GME: Graduate Medical Education
REDCap: Research Electronic Data Capture
WFUSOM: Wake Forest University School of Medicine
