# Supplementary material for: Local Culture and Community Through a Digital Lens: Viewpoint on Designing and Implementing a Virtual Second Look Event for Residency Applicants
Source: JMIR Med Educ. 2023 Sep 11;9:e44240. doi: 10.2196/44240 (PMC10520764; doi:10.2196/44240)
Supplement: Multimedia Appendix 1 [file mededu_v9i1e44240_app1.docx]

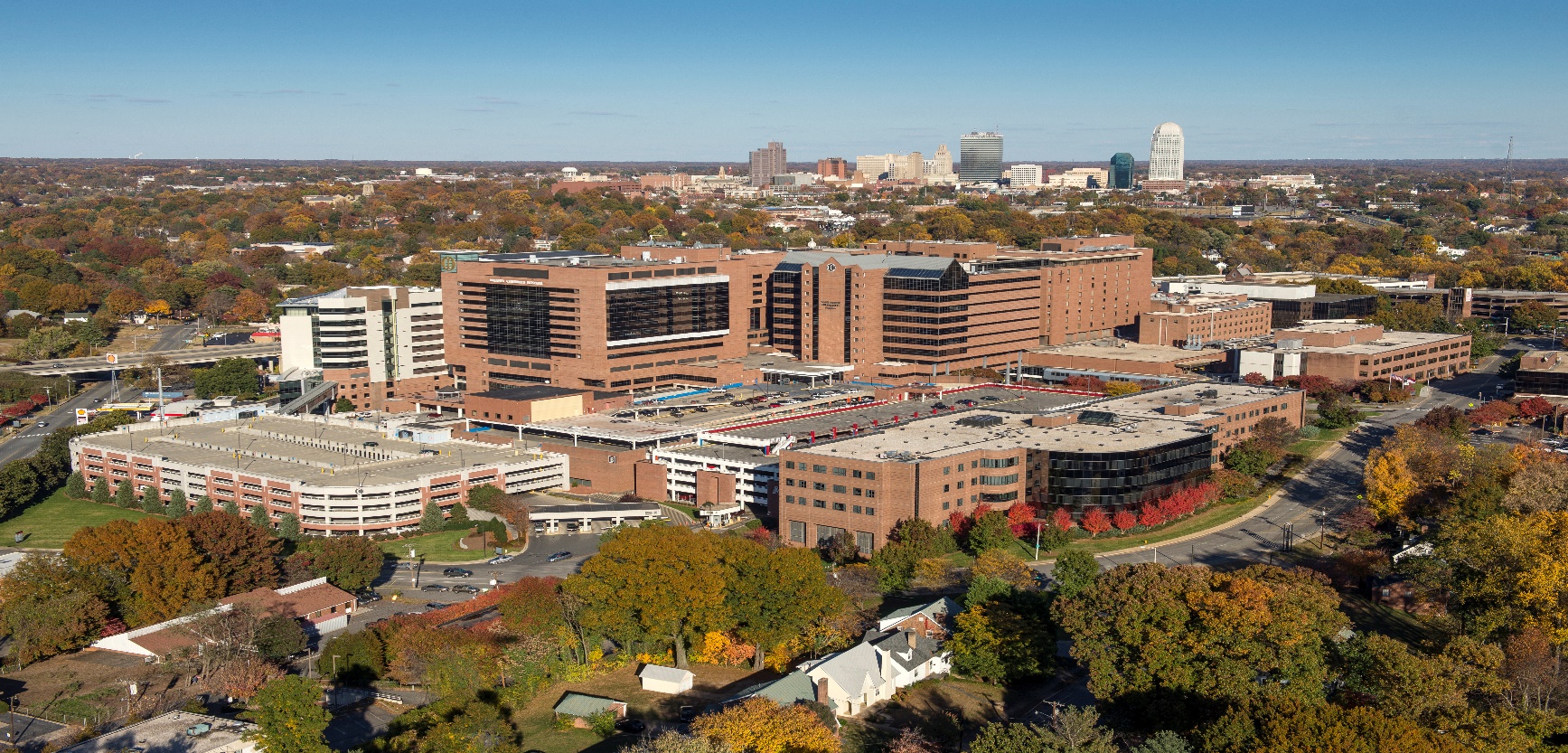


**4th Annual GME Wide Diversity Event**

February 12, 2022: Itinerary

- **10:00-10:10 am: Welcome and Brief Introductions from the Diversity Event Co-Chairs -**Dr. Amber Brooks and Dr. Nancy Denizard-Thompson
- **10:10-10:15 am: Greeting from Julie Freischlag,** **MD, FACS, FRCSEd (Hon), DFSVS, CEO of Atrium Health Wake Forest Baptist, Dean of Wake Forest School of Medicine, and Chief Academic Officer of Atrium Health Enterprise.**
- **10:15-10:20 am: Greeting from Mayor of Winston-Salem -** Allen Joines
- **10:20-10:25 am: Innovation and Life in Winston Salem**
- **10:25-10:50 am: Key Note Speaker: Gregory Townsend, MD,** Associate Dean for Diversity and Inclusion, UVA Health
- **10:50-11:20 am: Q&A with Institutional Leadership**
  - **Kevin High, MD, President of the Wake Forest Baptist Health System-** Clinical Mission
  - **Goldie Byrd, PhD, Director, Maya Angelou Center for Health Equity-** Research/Community Engagement Mission
  - **Brenda Latham-Sadler, MD, Senior Associate Dean for Justice Equity, Diversity and Inclusion for Academic Enterprise-** Diversity and Inclusion Mission
  - **Mitchell Sokolosky, MD, Associate Dean, GME-** Education Mission
- **11:20-11:50 am: Departmental Break Out Sessions -**Join a program’s Breakout session
- **11:50-12:30 pm: Resident Q&A: “Life as a Resident at Atrium Health Wake Forest Baptist”**
