# Supplementary material for: Local Culture and Community Through a Digital Lens: Viewpoint on Designing and Implementing a Virtual Second Look Event for Residency Applicants
Source: JMIR Med Educ. 2023 Sep 11;9:e44240. doi: 10.2196/44240 (PMC10520764; doi:10.2196/44240)
Supplement: Multimedia Appendix 2 [file mededu_v9i1e44240_app2.pdf]

Pre-Event Registration

We are looking forward to your participation in the virtual Atrium Wake Forest Baptist Health Virtual Second Look on Friday, February 18, 2021 from 12p-2p EST.

Following registration confirmation, you will be emailed a zoom meeting link to the email address provided in this survey closer to the date. Please take a few minutes to complete the survey to register for the event. Your answers will help us better prepare for your virtual experience. All answers will be kept confidential. Please be reassured that your placement on the rank list will not be influenced by your answers to these questions.

First name: ______________________________

Last name: ______________________________

Email address to receive event link: ______________________________

Please indicate your age range:

- ◯ 20-30
- ◯ 31-40
- ◯ 41-50
- ◯ 51+
- ◯ Prefer not to say

Please indicate your race (select one or more):

- ☐ American Indian or Alaska Native
- ☐ Asian
- ☐ Black or African American
- ☐ Native Hawaiian or Other Pacific Islander
- ☐ White
- ☐ I do not wish to disclose this information

Please indicate your ethnicity:

- ◯ Hispanic or Latino
- ◯ Not Hispanic or Latino
- ◯ Not Specified
- ◯ I do not wish to disclose this information

Please indicate the residency you are applying for:

- ◯ Anesthesiology
- ◯ Child Neurology
- ◯ Dermatology
- ◯ Diagnostic Radiology
- ◯ Emergency Medicine
- ◯ Family Medicine
- ◯ General Surgery
- ◯ Internal Medicine
- ◯ Interventional Radiology
- ◯ Neurology
- ◯ Neurosurgery
- ◯ Obstetrics/Gynecology
- ◯ Ophthalmology
- ◯ Orthopedic Surgery
- ◯ Otolaryngology
- ◯ Pathology
- ◯ Pediatrics
- ◯ Plastic Reconstructive Surgery
- ◯ Psychiatry
- ◯ Radiation Oncology
- ◯ Urology
- ◯ Other

Please indicate other program:	__________________________________
Please indicate your goal(s) for attending the Atrium Health Wake Forest Baptist GME virtual second look (check all that apply):	☐ Networking with other resident applicants<br>☐ Learning more living in the Winston-Salem area<br>☐ Learning more about raising a family in the Winston-Salem community<br>☐ Learning more the experience and culture of Winston-Salem<br>☐ Learning more about arts and music scene in Winston-Salem<br>☐ Learning more about the restaurant and bar/wineries in Winston-Salem<br>☐ Learning more about sports and outdoor activities in Winston-Salem<br>☐ Interacting with leaders in the Wake Forest School of Medicine and/or Atrium Health Wake Forest Baptist system<br>☐ Learning from residents currently in training on their experience<br>☐ Other
Please tell us more about other goals:	____
How did you hear about this event? (check all that apply)	☐ ERAS communication<br>☐ Social media<br>☐ Email<br>☐ Word-of-mouth<br>☐ Other
Please elaborate on other:	____
Do you wish to receive a complimentary packet about Winston-Salem mailed to your home?	◯ Yes<br>◯ No
Please provide your street address:	__________________________________
City:	__________________________________

State:

- ◯ Alabama
- ◯ Alaska
- ◯ Arizona
- ◯ Arkansas
- ◯ California
- ◯ Colorado
- ◯ Connecticut
- ◯ Delaware
- ◯ Florida
- ◯ Georgia
- ◯ Hawaii
- ◯ Idaho
- ◯ Illinois
- ◯ Indiana
- ◯ Iowa
- ◯ Kansas
- ◯ Kentucky
- ◯ Louisiana
- ◯ Maine
- ◯ Maryland
- ◯ Massachusetts
- ◯ Michigan
- ◯ Minnesota
- ◯ Mississippi
- ◯ Missouri
- ◯ Montana
- ◯ Nebraska
- ◯ Nevada
- ◯ New Hampshire
- ◯ New Jersey
- ◯ New Mexico
- ◯ New York
- ◯ North Carolina
- ◯ North Dakota
- ◯ Ohio
- ◯ Oklahoma
- ◯ Oregon
- ◯ Pennsylvania
- ◯ Rhode Island
- ◯ South Carolina
- ◯ South Dakota
- ◯ Tennessee
- ◯ Texas
- ◯ Utah
- ◯ Vermont
- ◯ Virginia
- ◯ Washington
- ◯ West Virginia
- ◯ Wisconsin
- ◯ Wyoming

Zipcode: ______________________________

Do you consent to us using your de-identified data for future event planning and research purposes? This will not affect your ability to register or participate in the event.

- ◯ Yes
- ◯ No
