# Supplementary material for: Local Culture and Community Through a Digital Lens: Viewpoint on Designing and Implementing a Virtual Second Look Event for Residency Applicants
Source: JMIR Med Educ. 2023 Sep 11;9:e44240. doi: 10.2196/44240 (PMC10520764; doi:10.2196/44240)
Supplement: Multimedia Appendix 3 [file mededu_v9i1e44240_app3.pdf]

Post-Event Survey

Thank you [first_name] for attending the Atrium Health Wake Forest Bapist Virtual 2nd look GME event. We really appreciate you joining our event!

Please take moment to complete this survey to help us understand how to improve this event going forward.

Thank you!

Please rate the following aspects of the Welcome Session Presentation with:

Lauren Strauss, DO, PD Child Neurology
Mitch Sokolosky, MD, AHWFB Designated Institutional Official
Marcheta Cole Keefer, VisitWS organization
Jacki Martindale, DO, Assoc. PD Child Neurology

		Strongly Agree	Agree	Neutral	Disagree	Strongly Disagree
1)	The presenter was enthusiastic and stimulated my interest	○	○	○	○	○
2)	The program was clear and organized	○	○	○	○	○
3)	The program met my expectations	○	○	○	○	○
4)	The session was valuable to me	○	○	○	○	○
5)	New information was gained that I did not get during my interview day	○	○	○	○	○

Please rate the following aspects of the Session 1: Living in Winston Salem/ Raising a Family in Our Community

		Strongly Agree	Agree	Neutral	Disagree	Strongly Disagree
6)	The presenters were enthusiastic and stimulated my interest	○	○	○	○	○
7)	The program was clear and organized	○	○	○	○	○
8)	The program met my expectations	○	○	○	○	○
9)	The session was valuable to me	○	○	○	○	○
10)	New information was gained that I did not get during my interview day	○	○	○	○	○

Please rate the following aspects of Session 2: Experiencing Winston Salem Arts and Music

		Strongly Agree	Agree	Neutral	Disagree	Strongly Disagree
11)	The presenters were enthusiastic and stimulated my interest	◯	◯	◯	◯	◯
12)	The program was clear and organized	◯	◯	◯	◯	◯
13)	The program met my expectations	◯	◯	◯	◯	◯
14)	The session was valuable to me	◯	◯	◯	◯	◯
15)	New information was gained that I did not get during my interview day	◯	◯	◯	◯	◯

Please rate the following aspects of Session 3: Where to Eat and Drink like a Local (Food/Wine/Beer)

		Strongly Agree	Agree	Neutral	Disagree	Strongly Disagree
16)	The presenters were enthusiastic and stimulated my interest	◯	◯	◯	◯	◯
17)	The program was clear and organized	◯	◯	◯	◯	◯
18)	The program met my expectations	◯	◯	◯	◯	◯
19)	The session was valuable to me	◯	◯	◯	◯	◯
20)	New information was gained that I did not get during my interview day	◯	◯	◯	◯	◯

Please rate the following aspects of Session 4: Enjoying Sports and the Outdoors

		Strongly Agree	Agree	Neutral	Disagree	Strongly Disagree
21)	The presenters were enthusiastic and stimulated my interest	◯	◯	◯	◯	◯
22)	The program was clear and organized	◯	◯	◯	◯	◯
23)	The program met my expectations	◯	◯	◯	◯	◯
24)	The session was valuable to me	◯	◯	◯	◯	◯
25)						

	Strongly Agree	Agree	Neutral	Disagree	Strongly Disagree
New information was gained that I did not get during my interview day	○	○	○	○	○

Please rate the OVERALL program

	Strongly Agree	Agree	Neutral	Disagree	Strongly Disagree
26) Overall, the program was well organized	○	○	○	○	○
27) This virtual event met my expectations/goals	○	○	○	○	○

28) Please list the top 3 highlights of the virtual 2nd look event:

____

29) How can we improve this virtual 2nd look experience going forward?

____

30) Please rate the overall effectiveness of the 2nd look event:

Not effective — Neutral — Very effective

(Place a mark on the scale above)

31) Please include any additional thoughts, comments or feedback:

____
